# Effects of Participating in a Research Project During the COVID-19 Pandemic on Medical Students’ Educational Routines and Mental Health: Protocol for a Web-Based Survey Study

**DOI:** 10.2196/24617

**Published:** 2021-04-09

**Authors:** Débora Cerqueira Calderaro, Barbara Stadler Kahlow, Gabriela Araújo Munhoz, Samuel Elias Basualto Dias, João Vitor Ziroldo Lopes, Aline Rizzo Borges, Henrique De Ataíde Mariz, Kirla Wagner Poti Gomes, Lilian David De Azevedo Valadares, Nafice Costa Araújo, Sandra Lucia Euzébio Ribeiro, Adriana Maria Kakehasi, Ana Paula Monteiro Gomides Reis, Cláudia Marques, Edgard Torres Reis-Neto, Eduardo Dos Santos Paiva, Gecilmara Salviato Pileggi, Gilda Aparecida Ferreira, José Roberto Provenza, Licia Maria Henrique Mota, Ricardo Machado Xavier, Maycoln Leôni Martins Teodoro, Marcelo De Medeiros Pinheiro, The Voluntary Brazilian Society Of Rheumatology Task Force Against COVID-19

**Affiliations:** 1 Universidade Federal de Minas Gerais Belo Horizonte Brazil; 2 Rheumatology Department Hospital Universitário Evangélico Mackenzie Paraná Brazil; 3 Rheumatology Department Santa Casa de Misericórdia de São Paulo São Paulo Brazil; 4 Rheumatology Department Universidade Federal do Amazonas Manaus Brazil; 5 Medical School Universidade de São Paulo São Paulo Brazil; 6 Medical School Centro Universitário de Brasília Brasília Brazil; 7 Rheumatology Department Universidade Federal de Pernambuco Recife Brazil; 8 Rheumatology Department Universidade Federal de Fortaleza Fortaleza Brazil; 9 Rheumatology Department Hospital do Servidor Público Estadual Instituto de Assistência Médica ao Servidor Público Estadual São Paulo Brazil; 10 Rheumatology Department Universidade de Brasília Brasília Brazil; 11 Rheumatology Department Universidade Federal de São Paulo São Paulo Brazil; 12 Rheumatology Department Universidade Federal do Paraná Curitiba Brazil; 13 Rheumatology Department Faculdade de Ciências da Saúde de Barretos Barretos Brazil; 14 Rheumatology Department Pontifícia Universidade Católica de Campinas Campinas Brazil; 15 Rheumatology Department Universidade Federal do Rio Grande do Sul Porto Alegre Brazil; 16 see Acknowledgments

**Keywords:** SARS-CoV-2, COVID-19, medical education, observational, cross-sectional, case-control study, voluntary, mental health, rheumatic disease, medical student, protocol, survey

## Abstract

**Background:**

The COVID-19 pandemic has resulted in social isolation, which has a potential negative impact on the educational routines (eg, the suspension of face-to-face appointments) and mental health of medical students. The Mario Pinotti II (MPII) study is a 24-week observational study that conducted scheduled telephone calls every 2 weeks to verify the occurrence of COVID-19 in patients with rheumatic diseases on chronic hydroxychloroquine therapy (from March 29, 2020, to September 30, 2020). The effects of voluntarily participating in a research project (ie, one that involves interactions via telephone contact with patients, professors, rheumatologists, and colleagues) on the daily lives and mental health of medical students requires evaluation.

**Objective:**

As medical students are professionals in training and have a high level of responsibility in terms of handling the emotional and physical aspects of several diseases, this study aims to evaluate the impacts of the COVID-19 pandemic and participation in the MPII study on the educational routines and mental health of medical students.

**Methods:**

A web-based survey was carried out to perform a cross-sectional comparative assessment of medical students who participated in the MPII study and their colleagues who were not involved in the MPII study. Participants from both groups were matched based on sex, age, and medical school. The web questionnaire was developed by a panel composed of graduate medical students, rheumatologists, medical school professors, and a psychology professor. The questionnaire included details on demographic and life habits data and evaluated participants' impressions of the MPII study and the impact of the COVID-19 pandemic on their educational routines and medical training. In addition, depression, anxiety, and stress were evaluated using the Brazilian version of the Depression, Anxiety, and Stress Scale (DASS)-21, and currently, the DASS-21 scores are grouped as those that indicate a low, moderate, or high risk of mental distress. This project was approved by the Federal University of São Paulo Ethics Committee (CAAE: 34034620.0.0000.5505).

**Results:**

Data were collected from both medical student groups from July 20 to August 31, 2020. Data extraction was completed in September 2020. The data analysis is ongoing. We expect the results to be published in the first semester of 2021.

**Conclusions:**

This study will provide insight into the effects of participating in a research project on depression, anxiety, and stress, which will be determined by applying the DASS-21 to a large sample of Brazilian undergraduate medical students. We will also evaluate the impact of the COVID-19 pandemic on medical students’ educational routines and medical training.

**International Registered Report Identifier (IRRID):**

DERR1-10.2196/24617

## Introduction

In December 2019, a respiratory disease (COVID-19) that is caused by the novel SARS-CoV-2 was identified in Wuhan City, Hubei Province, China. Several weeks later, the World Health Organization declared COVID-19 as an international public health emergency and pandemic [1].

Given the high levels of community transmission of SARS-CoV-2, several approaches have been recommended to mitigate global viral spread; social distancing, quarantine, intermittent hand hygiene, and universal masking are especially important approaches. However, these government recommendations vary according to each country and have resulted in home isolation, fear, uncertainty, anxiety, depression, high alcohol intake, domestic violence, education impairment, and severe economic burdens [2-4].

With regard to medical education, the majority of medical classes and face-to-face practices were suspended for a long period while medical schools prepared for remote education. Furthermore, several students have been invited to work on the front line of the pandemic (ie, treating patients with COVID-19)), which allowed them to graduate earlier [5,6]. These measures, which are associated with remote (not in-person) training, have hampered medical training for working with patients and other relevant aspects, including hospital and outpatient clinics and regulatory processes [7]. Several researchers have highlighted a gradual increase in anxiety levels among medical students during the COVID-19 pandemic, suggesting that the pandemic may be impairing several aspects of social relationships, technical performance, and mental health [7,8].

It is worth emphasizing that medical training is often exhausting due to specific technical requirements and a large number of stressful factors [9], including full-time dedication, personal life–related effort and sacrifice, close contact with severe diseases and death, and physical and emotional distress [10]. During the COVID-19 pandemic, extensive and intense workloads, difficulties in reconciling personal life with studies, competitiveness, sleep deprivation, fears of making mistakes and getting sick, tiredness, and decision making under pressure may result in high levels of anxiety and depression in medical students [9-13].

European studies have reported that around 30% of medical students experience some level of depression or anxiety. In Brazil, studies have suggested that 20%-50% of medical students experience mood changes [12]. In addition, depression and suicidal ideation rates are higher in medical students than in the general population, and these students generally seek less help from psychological or psychiatric professionals [5-12]. Several psychiatric illnesses and personality disturbances have been reported to be related to such behavior, including eating disorders, the denial of reality, alcoholism, the abuse of illicit drugs, a lack of commitment, obsessive-compulsive disorder, anxiety, depression, and increased suicide rates [7]. Thus, medical students are susceptible to experiencing inadequate or nonadaptive responses to emotional distress [7,13].

Since January 2020, the World Health Organization has been warning the public that the COVID-19 pandemic is generating stress in the general population [1], especially stress related to uncertainties about the course and prognosis of the disease; fear; a lack of resources for diagnosis and treatment; a shortage of food, medication, and adequate supplies of personal protective equipment for health care professionals; feelings of missing the freedom of travel; and conflicting information delivered by governmental authorities or social media [14-16]. It has also been reported that the incidence of several psychological disorders increased during previous pandemics, including the SARS (Severe Acute Respiratory Syndrome and MERS (Middle Eastern Respiratory Syndrome) pandemics. Such disorders mainly included anxiety and depression [8].

During the COVID-19 pandemic in China, a study that evaluated 217 medical students reported that depression and anxiety occurred in 35% and 22% of students, respectively [8]. Furthermore, researchers at medical schools in Wisconsin believe that students are great allies of doctors [7].

The Mario Pinotti II (MPII) study is a noninterventional, observational, multicenter, parallel-group cohort study that included adult volunteers (aged ≥18 years) with a previously known diagnosis of rheumatic disease. These participants were on hydroxychloroquine for at least 30 days prior to baseline. The MPII is a 24-week prospective study that included more than 10,000 individuals from 20 centers in Brazil. A total of 6 sequential telephone calls were scheduled during the community transmission of SARS-CoV-2. These calls were performed by 395 volunteer medical students [17].

Given the close social interaction between the patients and controls, which was facilitated through periodic telephone contact, as well as the social interactions among principal investigators, study coordinators, and professors, our main hypothesis was that medical students who participated in the MPII study would experience less emotional distress than their colleagues who did not participate in the MPII study. [12-15].

Our objectives were to evaluate the impact of participating in the MPII study on the mental health (evaluated using the Depression, Anxiety, and Stress Scale [DASS]-21) [18,19], professional improvement, and commitment perceptions of medical students during the COVID-19 pandemic. We also aimed to identify potential impairments in the educational routines of students’ medical schools and to report on COVID-19 diagnoses among this population.

## Methods

### Study Design

We will conduct a comparative, cross-sectional, observational, case-control study that used a voluntary web-based survey. The survey was conducted according to the Checklist for Reporting Results of Internet E-Surveys (CHERRIES) statement [20].

### Study Population

Medical students who were involved in the MPII study (volunteer group) and their colleagues who were not involved in the MPII study (control group) [17] were recruited.

### Sample Size

Convenience sampling was conducted based on voluntary involvement, as per the investigators in the MPII Study. Of the 20 MPII centers, 14 (70%) participated in this study. All students who successfully answered the web questionnaire during the data collection period were included in this study.

### Inclusion Criteria

The volunteer group consisted of medical students who participated as volunteers in the MPII study and were aged ≥18 years. The control group consisted of medical students who did not participate in the MPII study. For each participant in the volunteer group, at least two other students were enrolled as controls. This was done to ensure that the number of participants was sufficient for stratified analysis, as we expected that the prevalence of mental health issues among medical students would be high [21].

An electronic informed consent form was provided to both groups, and participants were required to sign it before they were granted access to the survey questionnaire.

### Exclusion Criteria

Participants were excluded if they refused to provide informed consent or withdrew their consent.

### Survey Questionnaire

This study was approved by the Federal University of São Paulo Ethics Committee (Certificado de Apresentação de Apreciação ética [CAAE]: 34034620.0.0000.5505) on July 13, 2020.

The complete survey questionnaire form can be found in Multimedia Appendix 1. It includes 69 questions. The time required for completing the survey was 20 minutes. The questionnaire was developed and provided to participants in Portuguese (their mother language), and it was only translated in order to be published. The translation was verified by an experienced translator in Brazil.

A panel of undergraduate medical students, rheumatologists, and medical school professors who were involved in the MPII study, as well as a psychology professor who had experience in conducting web-based surveys to evaluate the mental health of medical students and health care professionals, were responsible for developing the web-based survey questionnaire. The students in the panel that developed the survey (n=3) tested the questionnaire. Afterward, it was distributed to the other participants of this study.

The volunteer group received an invitation video that provided explanations about the survey and the link to access the web questionnaire. Subsequently, the volunteers were requested to send an invitation link to colleagues who were not participating in the MPII study. These colleagues were added to the control group. No identification data were requested.

An informed consent form was integrated into the web questionnaire. Participants were required to provide their consent to participate in the survey (electronic informed consent) before accessing the questionnaire of the study or providing any information.

Participants can provide an email address if they want to receive their mental health evaluation results. Participants or researchers who require a psychological or psychiatric evaluation will be provided (through email contact) with guidance on accessing local facilities that offer these services. All participants were informed that providing an email address is voluntary and can be done before answering the survey questionnaire.

Demographic and epidemiologic data and details about comorbidities, life habits (smoking, alcohol intake, illicit drug use, and physical activity), and concomitant medications were recorded. In addition, specific aspects related to medical schools, such as the type of school (public or private), costs, teaching activities, and feelings about medical training during the pandemic, were addressed. With regard to participants in the volunteer group, their impressions of the procedures in the MPII study and the study’s impact on their daily routines were evaluated.

The Brazilian version of the DASS-21 was used to evaluate mental health [18,19]. The DASS-21 is a set of three self-report scales with seven items each. The items were designed to measure emotional status. The depression domain assesses dysphoria, hopelessness, the devaluation of life, self-deprecation, a lack of interest or commitment, anhedonia, and inertia. The anxiety domain evaluates autonomic symptoms, skeletal muscle effects, coping, and experiences. The stress domain is sensitive to levels of chronic nonspecific arousal, and it assesses difficulties in relaxing; nervous arousal; and the state of being easily upset/agitated, irritable/overreactive, and impatient. Scores for depression, anxiety, and stress are calculated by summing the scores of the relevant items. The DASS-21 is based on a dimensional concept of psychological disorders instead of a categorical concept of psychological disorders, and it was developed by accounting for the differences among the depression, anxiety, and stress experienced by subjects. Therefore, the scale does not have any direct implications for diagnosis.

DASS-21 scores were grouped as those that indicate a low, moderate, or high risk or mental distress. These will be stratified according to gender and the SD from the study population’s mean scores (low: lower than mean+1 SD; moderate: ranges from mean+1 SD to mean+2 SDs; high: greater than mean+2 SDs).

Recommended cutoff scores for conventional severity labels of depression, anxiety, and stress (normal, mild, moderate, severe, and extremely severe) were multiplied by 2, as shown in Table 1 [19].

**Table 1:** Cutoff scores for depression, anxiety, and stress according to the Depression, Anxiety, and Stress Scale (DASS)-21.

Severity label	Doubled DASS-21 domain scores [19]		
	Depression	Anxiety	Stress
Normal	0-9	0-7	0-14
Mild	10-13	8-9	15-18
Moderate	14-20	10-14	19-25
Severe	21-27	15-19	26-33
Extremely severe	≥28	≥20	≥34

### Data Collection

Data collection was performed by using a web questionnaire that was generated on the Google Forms platform. The questionnaire was disclosed to the research subjects via email and WhatsApp (WhatsApp LLC). The data collection period was from July 20 to August 31, 2020.

### Statistical Analysis

Descriptive analysis will be performed using absolute and relative frequencies for categorical variables and quantitative measures (means, quartiles, minimums, maximums, and SDs) for numerical variables. The normality of numerical variables will be evaluated using the Kolmogorov-Smirnov test. Numerical variables with normal distributions will be described as mean (SD), and nonnormal numerical variables will be described as median (IQR) or range (minimum-maximum).

The Chi-square association test with adjusted standardized residuals will be used to assess the association between categorical variables; the Fischer exact test will be used for small samples. The linear associations between two numerical variables will be evaluated using the Pearson or Spearman correlation method.

The comparison between the mean numerical variables with normal distributions in the volunteer group and those in the control group will be conducted by using the Student *t* test. If the assumption of normality is violated, the Mann-Whitney nonparametric test will be used.

Adjusted multiple linear regression models will be used to assess the simultaneous effects of sex, age, comorbidities, concomitant medications, and other confounding variables based on group type and predefined outcomes (anxiety, depression, and stress scores from the DASS-21). For dichotomous dependent variables, a logistic regression model will be used.

SPSS, version 20 (IBM Corporation) will be used for all analyses. A *P* value of <.05 will be considered significant.

## Results

Data were collected from both medical student groups from July 20 to August 31, 2020. Data extraction was completed on September 2020. The data analysis is ongoing. We expect the results to be published in the first semester of 2021.

## Discussion

This study has an unprecedented design, as it includes a very large sample of volunteer medical students from 14 Brazilian tertiary rheumatology centers. These students are currently monitoring the outcomes of 9589 patients with rheumatic diseases on hydroxychloroquine and assessing patients’ susceptibility to SARS-CoV-2 infection.

The main objective of this study is to evaluate the impact of participating in a research project during the COVID-19 outbreak on the mental health and learning behaviors of medical students. These variables were measured by using a structured web questionnaire about students’ volunteer participation in the MPII study and their ability to work with patients and professors in a real-life scenario.

This study has several innovative aspects, such as (1) the evaluation of depression, anxiety, and stress by applying the DASS-21 to a large sample of medical students and a control group; (2) medical students' impressions regarding the handling of the uncertainty and doubts of patients with rheumatic diseases, including the fear of illness, fear of dying, and shortage of medication during the outbreak; (3) the measurement of the impact of the COVID-19 pandemic on students’ educational routines and medical training; and (4) the fact that the web questionnaire was developed by a panel composed of graduate medical students, rheumatologists, medical school professors, and a psychology professor.

## Appendix

Multimedia Appendix 1 Survey instrument.

## Abbreviations

CHERRIES: Checklist for Reporting Results of Internet E-Surveys
DASS: Depression, Anxiety, and Stress Scale
MPII: Mario Pinotti II
MERS: Middle Eastern Respiratory Syndrome
SARS: Severe Acute Respiratory Syndrome
