# Supplementary material for: Effects of Participating in a Research Project During the COVID-19 Pandemic on Medical Students’ Educational Routines and Mental Health: Protocol for a Web-Based Survey Study
Source: JMIR Res Protoc. 2021 Apr 9;10(4):e24617. doi: 10.2196/24617 (PMC8041051; doi:10.2196/24617)
Supplement: Multimedia Appendix 1 [file resprot_v10i4e24617_app1.pdf]

Appendix

Survey questinnaire

1. Age (years):
2. Date of inclusion: __/__/__ (day/month/year)
3. Color

   White ( )

   Black ( )

   Asian ( )

   Mixed-race ( )

   Indigenous ( )

4. Gender

   Female ( )

   Male ( )

5. Marital Status

   Single ( )

   Married ( )

   Divorced ( )

   Widowed( )

6. Do you smoke or consume tobacco in other forms (electronic cigarette, hookah, others)?

   Yes ( )

   No ( )

   If “yes”, your consumption DURING QUARANTINE:

   Decreased ( )

   Increased ( )

   Stayed the same ( )

7. Alcohol intake PRIOR TO QUARANTINE [consider alcoholism if: 15 doses per week for men and 10 doses per week for women; one dose being equivalent to 285 mL of beer, 120 mL of wine, 30 mL of distilled beverage - whisky, vodka, sugarcane liquor [cachaça]).

   Yes ( ) Dose/week: ____________

No ( )

If “yes”, your alcohol intake DURING QUARANTINE:
Decreased ( )
Increased ( )
Stayed the same ( )

8. Do you use any licit or illicit drugs - not including alcohol or tobacco - for recreational purposes?
Yes ( )
No ( )

If “yes”, your use DURING QUARANTINE:
Decreased ( )
Increased ( )
Stayed the same ( )

9. Physical activity PRIOR TO QUARANTINE [“yes” being at least 150 minutes/week of moderate physical activity]
Yes ( )
No ( )

If “yes”, your physical activity DURING QUARANTINE:
Decreased ( )
Increased ( )
Stayed the same ( )

10. Do you live with your family ?
Yes ( ) No ( )

DURING THE COVID-19 PANDEMIC, are you with your family?
Yes ( ) No ( )

11. Do you have any comorbidity?
Hypertension ( )
Diabetes Mellitus ( )

Heart disease ( )

Kidney disease ( )

Dyslipidemia ( )

Overweight/Obesity ( )

Lung disease ( )

Thyroidopathy ( )

Depression ( )

Anxiety ( )

Obsessive-Compulsive Disorder ( )

Panic syndrome ( )

None ( )

Other: ____________________________

12. Do you take any medications daily?

Yes ( ) No ( )

Which: __________________

13. Do you have a previous academic degree?

Yes ( ) No ( )

Which: __________________

14. The college where you study is:

Private ( )

Public ( )

City: _________________________

State: [List for selection: AC, AL, AP, AM, BA, CE, ES, GO, MA MT, MS, MG, PA, PB, PR, PE, PI, RJ, RN, RS, RO, RR, SC, SP, SE, TO, DF]

15. If you study in a private college, are you having any difficulties in keeping your payments up to date?

Yes ( ) No ( )

16. Which period/semester of medical school are you currently attending?

[List from 1- 12] period/semester

17. Do you receive any institutional financial support?

( ) Direct costs (monthly charges)

( ) Indirect costs (feeding, habitation, study supplies)

( ) I do not receive any institutional support

If "yes", DURING QUARANTINE is this:

( ) Sustained

( ) Partially sustained

( ) Suspended

18. During the COVID-19 pandemic, in your medical school [select all alternatives that apply for your case]

( ) In-person classes were completely suspended

( ) In-person classes were switched to virtual classes

( ) Practical classes/Internship were kept as normal

( ) Practical classes/Internships were maintained, but changing the place where they were conducted

If in-person activities were maintained, in which healthcare unities? [select all alternatives that apply in your case]

Primary Care Health Unit ( )

Non-COVID-19 attending Unit ( )

COVID-19 attending Unit ( )

19. Regarding distance learning, I believe that learning was impaired

Totally Agree ( )

Agree ( )

Neutral ( )

Disagree ( )

Totally disagree ( )

20. Because of the quarantine/pandemic, I'm afraid that my medical training will be delayed

Totally Agree ( )

Agree ( )

Neutral ( )

Disagree ( )

Totally disagree ( )

21. Because of the quarantine/pandemic, I'm worried that my medical training will be impaired

    Totally Agree ( )

    Agree ( )

    Neutral ( )

    Disagree ( )

    Totally disagree ( )

22. During the quarantine/pandemic, I have been able to keep my study routine

    Totally Agree ( )

    Agree ( )

    Neutral ( )

    Disagree ( )

    Totally disagree ( )

23. I'm worried about how class activities will be replaced after the quarantine

    Totally Agree ( )

    Agree ( )

    Neutral ( )

    Disagree ( )

    Totally disagree ( )

24. I'm worried about what my school tests will be like after quarantine

    Totally Agree ( )

    Agree ( )

    Neutral ( )

    Disagree ( )

    Totally disagree ( )

25. Regarding COVID-19 [Check all relevant alternatives]

    I'm not worried about it ( )

    I have/had COVID-19 ( )

I’m afraid of getting sick ( )

I’m afraid to spread the virus to other people ( )

I’m afraid that someone in my family/close friends will get it ( )

I’m afraid that someone in my family/close friends will die because of it ( )

I’m afraid of dying because of it ( )

26. Are you participating in the Mario Pinotti II study?

    Yes ( ) No ( )

27. Besides the Mario Pinotti II study, are you currently participating in other research projects?

    Yes ( ) No ( )

    If “yes”, how many:_____________

28. Have you ever participated in a telephone inquiry project?

    Yes ( ) No ( )

29. Did you have any experience with the REDCap platform?

    Yes ( ) No ( )

30. In the beginning of the study, your training was provided by:[select all that apply]

    Explanatory videos and tutorials ( )

    In-person training with the Principal Investigator of my center ( )

    Virtual training with the Principal Investigator of my center ( )

31. This training was important for my awareness regarding the study procedures:

    Totally Agree ( )

    Agree ( )

    Neutral ( )

    Disagree ( )

    Totally disagree ( )

32. How many hours/week do you dedicate to the Mario Pinotti II study?

    Less than 2 hours ( )

2-4 hours ( )
4-6 hours ( )
6-8 hours ( )
More than 8 hours ( )

33. Regarding your study activities, select the situation that applies to you
I'm not performing any study activities ( )
I'm performing study activities without any difficulties ( )
I'm performing study activities with some difficulties ( )
I'm performing study activities with great difficulties ( )

34. Select the alternatives that have made it difficult for you to develop your tasks in the Mario Pinotti II study [select all that apply to you]
Lack of computer at home ( )
Lack of Internet/ Internet connection not working properly ( )
Difficulties in communication with the study head advisor ( )
Difficulties in communication with potential patients for the study [wrong phone numbers, patients do not answer or do not want to participate] ( )
Shyness while speaking on the cell phone with the patient ( )
Medication shortage ( )
Difficulties in using the call-center ( )
Difficulties/Doubts in the questionnaire structure ( )
Difficulties/Doubts about REDCap ( )
Lack of time, because I have other work to do ( )
Personal/Family problems ( )
Lack of motivation, despondency, or psychiatric suffering ( )
Other health problems ( )
No difficulties ( )

35. Regarding doubts that occurred during the study activities
( ) They were not resolved
( ) Sometimes they were resolved
( ) They were always resolved
( ) There were no doubts

36. Regarding doubts about the study (activities or patient recommendations), select the options that apply:
( ) Interaction with the principal investigator of my center
( ) Interaction with other volunteer students of the study
( ) Reading the Newsletter
( ) Interaction with sub-investigator
( ) Interaction with study coordinators
( ) There were no doubts

37. Patient's doubts about their rheumatologic disease or about COVID-19 that came up during the telephone interaction motivated me to study to find the answers
Totally Agree ( )
Agree ( )
Neutral ( )
Disagree ( )
Totally disagree ( )

38. Select below if your center used any of the following methodologies for prior contact with the patient in order to increase patient receptivity to the study :
( ) No prior contact was made
( ) In-person contact
( ) Telephone contact prior to the study contact
( ) Whatsapp contact
( ) Social media
( ) Telemedicine
( ) E-mail
( ) Other: _____________

39. In your opinion, most of the patients:
( ) Were not receptive about their participation in the study
( ) Were receptive about their participation in the study

40. In your opinion, most of the household contacts:
( ) Were not receptive about their participation in the study
( ) Were receptive about their participation in the study

41. Were the scheduled telephone calls appropriate?

( ) Yes ( ) No

42. During the study, select the elements that impacted your life: [select all that apply]

( ) Routine establishment (lessons, interviews, study,...)

( ) Scientific knowledge of the theme

( ) Knowledge about how a scientific study works

( ) Feeling of being useful in the pandemic (care of patients that aren't receiving medical care at the moment)

( ) Loss of shyness while interacting with patients

( ) Increased contact with patients

( ) Frustrations regarding patient reality

( ) Increased stress/anxiety or irritability

( ) Responsibility with deadlines and completion of the information regarding your interview/visit

43. During the telephone call, select the problems/concerns reported by the patients that you identified:[select all that apply]

( ) I did not identify any problems

( ) Lack/difficulty of access to chloroquine/hydroxychloroquine

( ) Lack/difficulty of access to other medications for chronic use

( ) Higher risk of COVID-19

( ) Higher risk of dying from COVID-19

( ) Possibility of worsening the rheumatic disease

( ) Loneliness (lack of having someone to talk to or to live with)

( ) Lack/difficulty in accessing information provided by the attending physician/team of the patient

( ) Loss of the patient follow-up/treatment of rheumatic disease

( ) Risk of unemployment

44. During the COVID-19 pandemic, your sleep pattern:

( ) Is unchanged

( ) Is better

( ) Is worse

Please read each statement and check a number 0, 1, 2 or 3 which indicates how much the statement applied to you OVER THE PAST WEEK (DASS21 Scale).

45. I found it hard to wind down
    (0) Did not apply to me at all
    (1) Applied to me to some degree, or some of the time
    (2) Applied to me to a considerable degree, or a good part of time
    (3) Applied to me very much, or most of the time

46. I was aware of dryness of my mouth
    (0) Did not apply to me at all
    (1) Applied to me to some degree, or some of the time
    (2) Applied to me to a considerable degree, or a good part of time
    (3) Applied to me very much, or most of the time

47. I couldn't seem to experience any positive feelings at all
    (0) Did not apply to me at all
    (1) Applied to me to some degree, or some of the time
    (2) Applied to me to a considerable degree, or a good part of time
    (3) Applied to me very much, or most of the time

48. I experienced breathing difficulty (e.g., excessively rapid breathing, breathlessness in the absence of physical exertion)
    (0) Did not apply to me at all
    (1) Applied to me to some degree, or some of the time
    (2) Applied to me to a considerable degree, or a good part of time
    (3) Applied to me very much, or most of the time

49. I found it difficult to work up the initiative to do things
    (0) Did not apply to me at all
    (1) Applied to me to some degree, or some of the time
    (2) Applied to me to a considerable degree, or a good part of time
    (3) Applied to me very much, or most of the time

50. I tended to over-react

(0) Did not apply to me at all
(1) Applied to me to some degree, or some of the time
(2) Applied to me to a considerable degree, or a good part of time
(3) Applied to me very much, or most of the time

51. I experienced trembling (e.g., in the hands)
(0) Did not apply to me at all
(1) Applied to me to some degree, or some of the time
(2) Applied to me to a considerable degree, or a good part of time
(3) Applied to me very much, or most of the time

52. I felt that I was using a lot of nervous energy
(0) Did not apply to me at all
(1) Applied to me to some degree, or some of the time
(2) Applied to me to a considerable degree, or a good part of time
(3) Applied to me very much, or most of the time

53. I was worried about situations in which I might panic and make a fool of myself
(0) Did not apply to me at all
(1) Applied to me to some degree, or some of the time
(2) Applied to me to a considerable degree, or a good part of time
(3) Applied to me very much, or most of the time

54. I felt that I had nothing to look forward to
(0) Did not apply to me at all
(1) Applied to me to some degree, or some of the time
(2) Applied to me to a considerable degree, or a good part of time
(3) Applied to me very much, or most of the time

55. I found myself getting agitated
(0) Did not apply to me at all
(1) Applied to me to some degree, or some of the time
(2) Applied to me to a considerable degree, or a good part of time
(3) Applied to me very much, or most of the time

56. I found it difficult to relax

(0) Did not apply to me at all
(1) Applied to me to some degree, or some of the time
(2) Applied to me to a considerable degree, or a good part of time
(3) Applied to me very much, or most of the time

57. I felt down-hearted and blue
(0) Did not apply to me at all
(1) Applied to me to some degree, or some of the time
(2) Applied to me to a considerable degree, or a good part of time
(3) Applied to me very much, or most of the time

58. I was intolerant of anything that kept me from getting on with what I was doing
(0) Did not apply to me at all
(1) Applied to me to some degree, or some of the time
(2) Applied to me to a considerable degree, or a good part of time
(3) Applied to me very much, or most of the time

59. I felt I was close to panic
(0) Did not apply to me at all
(1) Applied to me to some degree, or some of the time
(2) Applied to me to a considerable degree, or a good part of time
(3) Applied to me very much, or most of the time

60. I was unable to become enthusiastic about anything
(0) Did not apply to me at all
(1) Applied to me to some degree, or some of the time
(2) Applied to me to a considerable degree, or a good part of time
(3) Applied to me very much, or most of the time

61. I felt I wasn't worth much as a person
(0) Did not apply to me at all
(1) Applied to me to some degree, or some of the time
(2) Applied to me to a considerable degree, or a good part of time
(3) Applied to me very much, or most of the time

62. I felt that I was rather touchy

(0) Did not apply to me at all
(1) Applied to me to some degree, or some of the time
(2) Applied to me to a considerable degree, or a good part of time
(3) Applied to me very much, or most of the time

63. I was aware of the action of my heart in the absence of physical exertion (e.g., sense of heart rate increase, heart missing a beat)
(0) Did not apply to me at all
(1) Applied to me to some degree, or some of the time
(2) Applied to me to a considerable degree, or a good part of time
(3) Applied to me very much, or most of the time

64. I felt scared without any good reason
(0) Did not apply to me at all
(1) Applied to me to some degree, or some of the time
(2) Applied to me to a considerable degree, or a good part of time
(3) Applied to me very much, or most of the time

65. I felt that life was meaningless
(0) Did not apply to me at all
(1) Applied to me to some degree, or some of the time
(2) Applied to me to a considerable degree, or a good part of time
(3) Applied to me very much, or most of the time

66. Have you had symptoms suggestive of COVID19?
( ) Yes
( ) No

If yes, they were solved:
( ) At home, without hospitalization
( ) I went to the emergency room and was released without major severity
( ) Hospitalization
( ) Hospitalization in ICU
( ) Needed mechanical ventilation

67. Was it confirmed by a laboratory test (PCR in nasopharyngeal swab, serology, rapid test)?
( ) Yes
( ) No

68. Did any household contact have a COVID19 diagnosis?
( ) Yes
( ) No

If yes, how was it solved?
( ) Stayed in social distancing, without hospitalization
( ) Went to the emergency room and was released without major severity
( ) Hospitalization
( ) Hospitalization in ICU
( ) Needed mechanical ventilation

69. If you had COVID19, your contact for the infection (transmission) was:
( ) At home/family circle
( ) At medical school
( ) In the hospital/health system
( ) In the community

Please, send the research link to at least three of your classmates who are not participating in the Mario Pinotti II project
